# LINC01410 leads the migration, invasion and EMT of bladder cancer cells by modulating miR-4319 / Snail1

**DOI:** 10.1186/s12935-021-02119-z

**Published:** 2021-08-14

**Authors:** Wei Guo, Qimei Gai, Yue Ma, Zhengfei Shan, Jitao Wu

**Affiliations:** 1 Department of Radiotherapy, The Affiliated Yantai Yuhuangding Hospital of Qingdao University, Yantai, 264000 Shandong People’s Republic of China; 2 Department of Vascular Surgery, The Affiliated Yantai Yuhuangding Hospital of Qingdao University, Yantai, 264000 Shandong People’s Republic of China; 3 Department of Urology, Mianyang Central Hospital, Mianyang, 621000 Sichuan People’s Republic of China; 4 Department of Organ Transplantation, The Affiliated Yantai Yuhuangding Hospital of Qingdao University, Yantai, 264000 Shandong People’s Republic of China; 5 Department of Urology, The Affiliated Yantai Yuhuangding Hospital of Qingdao University, Yantai, 264000 Shandong People’s Republic of China

**Keywords:** Bladder cancer, LINC01410, miR-4319, Snail1

## Abstract

**Background:**

Several previous studies have implied the significance of lncRNA1410 (LINC01410) in gastric cancer, rectal cancer, and cervical cancer. Nevertheless, the potential of LINC01410 in bladder cancer (BC) development has not been addressed.

**Methods:**

The related mechanisms were explored by qRT-PCR analysis, CCK-8 assay, cell transfection assay, Transwell assay, Western Blot analysis, Luciferase reporter assay and RNA pull-down assay.

**Results:**

In the following study, LINC01410, characterized as an oncogene, exhibited high levels of expression in BC tissues as compared to normal tissues and its expression leads to a reduced prognosis of BC. Functional characterization of LINC01410 showed that knocking down LINC01410 could markedly reduce the invasion and proliferation capacity of T24 and 5637 cells. Mechanistically, LINC01410 served as a sponge for miR-4319 and the findings were further attested through luciferase reporter assay. Analysis of miR-4319 demonstrated its low expression in BC tissues as compared to normal tissues and knocking down LINC01410 significantly increased miR-4319. Data obtained from rescue assay discovered that silencing of miR-4319 in T24 and 5637 cells restored the proliferation and invasion capacity of LINC01410.

**Conclusions:**

Taken together, this study is the first report on the oncogenic potential of LINC01410 in BC development by upregulating Snail1 protein and downregulating miR-4319.

*Trial registration* Retrospectively registered.

**Supplementary Information:**

The online version contains supplementary material available at 10.1186/s12935-021-02119-z.

## Introduction

Bladder cancer (BC) is the ninth most commonly identified cancer worldwide and one of the most malignant cancers in males [1, 2]. Genetic factor in BC development includes slow acetylation of *N*-acetyltransferase which is a vital enzyme in aromatic enzymes metabolism [3]. Whereas, other key factors include arsenic contamination in drinking water, long exposure to aromatic amines, and tobacco smoking [4, 5]. Although multiple mechanisms and advances have been developed in the treatment and diagnosis of BC, in the last few years the mortality and survival rate of patients have not been improved [6, 7]. Henceforth, it is inevitable to investigate the fundamental mechanisms of BC development to develop new biomarkers and strategies for the treatment of BC.

LncRNAs account for > 80% of genomic non-coding RNAs and usually have the length of several hundred nucleotides [8]. LncRNAs do not code for any protein, however, they possess several key biological functions, including cell migration, propagation, and development [9, 10]. In addition, these intergenic RNAs have been evaluated as effective therapeutic biomarkers in many researches [11]. Various non-coding RNAs are abnormally expressed in BC and their expression levels are associated with the clinico-pathological features of the patients. For example, Increased lncRNA ABHD11-AS1 represses the malignant phenotypes of BC [12], Tetracycline-inducible shRNA targeting antisense lncRNA HIF1A-AS2 represses the malignant phenotypes of BC [13]. Moreover, LncRNA PVT1 accelerates malignant phenotypes of BC cells by modulating miR-194-5p/BCLAF1 axis as a ceRNA [14].Nonetheless, most lncRNAs remain to be investigated and validated for their functional roles in BC.

Recently, several studies have demonstrated the high expression and oncogenic potential of long intergenic ncRNA 1410 (LINC01410) in gastric cancer, cervical cancer, and rectal cancer [15–20]. However, the significance of LINC01410 in BC development and metastasis has not been reported. The following study aimed to attest the role of LINC01410 in epithelial-to-mesenchymal transition (EMT) migratory and invasive abilities of BC, and miR-4319 as its potential target. The results indicated that LINC01410 is upregulated in BC tissue as compared to normal tissues but downregulates the expression levels of miR-4319. Furthermore, the following study also evaluated the low expression levels of miR-4319 in BC which also has not been published before. However, low expression levels of miR-4319 have been corroborated in liver and thyroid cancers [21–23]. Taken together, the study confirms the oncogenic role of LINC01410 in BC proliferation, migration, invasion, and EMT transformation by sponging miR-4319, documenting LINC01410 as a potential therapeutic target.

## Materials and methods

### Software used for analysis

Cancer Genome Atlas (TCGA, https://cancergenome.nih.gov/) database comprising 28 normal bladder tissues and 404 cancerous tissues was analyzed for evaluating the expression and prognosis of LINC01410. Target LINC01410 of miRNAs and mRNA were assessed through LncBase website. Correlation analysis was performed based on patient’s information with complete clinical data from TCGA.

### Patient selection

Sixty pairs of BC adjacent and normal tissue samples were collected for this study. Data of all individuals was collected following a brief interview, and written informed permission was obtained from each subject. All of the patients were diagnosed as transitional cell carcinomas by three independent pathologists. All the clinical procedures were approved by the ethical committee of The Affiliated Yantai Yuhuangding Hospital of Qingdao University in 2020 (approval number: [KY-E-2020–02-08]) and conformed well to the international guidelines.

### Quantitative real-time PCR (qRT-PCR) analysis

Total RNA was separated from the tumor tissues and control samples using RNA extraction kit (Invitrogen, Carlsbad, CA) according to the manufacturer’s protocol. The purity and quantity of isolated total RNA were evaluated using a spectrophotometer. Then, performing cDNA synthesis from 1 μg total RNA in 20 μL reaction volume using cDNA synthesis kit (Applied Biosystems, Foster City, CA) with random primer for following qRT-PCR using ABI7900HT Fast Real-time PCR machine. miRNA expression was evaluated using RT kit (GenePharma, Shanghai, China) and Script SYBR Green PCR kit in an ABI7900HT Fast Real-time PCR machine. GAPDH was the internal control for miRNA and gene expression was calculated with 2^−ΔΔCt^ method [24].

### Cell proliferation (CCK-8) assay

CCK-8 (Dojindo Laboratories, Kumamoto, Japan) method was conducted for proliferation analysis following the given guidelines. Concisely, cell suspension containing 1 × 10^3^ BC cells was loaded on assay plates and incubated for indicated time intervals. After incubation, cells were stained with 10 µL of CCK-8 solution and cultured in the dark for 0 h, 24 h, 48 h, 72 h and 96 h at 37 °C [18]. The absorbance at 450 nm was observed with a microplate reader (EL340; Bio‐Tek Instruments, Hopkinton, MA, USA).

### Cell transfection assay

BC cell lines (T24, J82, UMUC3, and 5637) and the normal cell line (SV-HU-1) were obtained from American Type Culture Collection (ATCC; Manassas, VA, USA). Cell culturing was done with RPMI-1640 culture medium comprising 10% FBS to incubate at 37 °C containing 5% CO_2_. siRNAs silencing LINC01410 for knockdown were purchased from (Genechem (Shanghai, China). Lipofectamine 2000 (Invitrogen, Carlsbad, CA, USA) was used for transfection according to the guidelines given by the manufacturer. The cells were collected after 24 h of transfection, and transfection efficiency was calculated through RT-qPCR analysis [15].

### Transwell assay

Transwell assay was performed to assess cell proliferation and migration [18]. The invasion ability of the cells was assessed with pre-coated Matrigel transwell chambers. Migration assay was conducted by culturing the cells in the upper chamber (8.0 μm pore size, Corning) with serum-free (BD Biosciences) medium. After 24 h incubation, the upper chamber was seeded with cells. Whereas, the lower chamber was implanted with complete medium. After 48 h of cell migration, the cells were fixed with paraformaldehyde and subsequently stained crystal violet. Cells were counted under the microscope (Nikon, Tokyo, Japan).

### Western blot analysis

For protein analysis, cells lysate was prepared RIPA lysis buffer containing proteases. Protein bioassay kit (Thermo Scientific Pierce, Carlsbad, USA) was used to detect protein concentrations. Proteins were separated with SDS-PAGE and transferred into PVDF, sealed with milk. Following this, membranes were cultured with primary antibodies overnight against Snail1 (ab53519, Abcam), E-cadherin (Ab194982, Abcam, Cambridge), N-cadherin (ab202030, Abcam), and Vimentin (ab24525, Abcam) to detect protein levels in T24 and 5637 cells. Chemiluminescence detection system (Clinx) was used to visualize the number of proteins [17].

### In-vivo assay for tumor angiogenesis

In-vivo assays were performed after approval of the Animal Ethics Committee of The Affiliated Yantai Yuhuangding Hospital of Qingdao University. Initially, cells were randomized and 1 × 10^7^ T24 cells (SH-NC, SH-LINC01410 #1) were used for subcutaneous injection into the mice models to develop xenografts. The size of subcutaneous tumor formation was measured every 7 days during the course of 7–28 days. Four weeks later, tumor samples from sacrificed mice were subjected to further analysis. To estimate tumor formation, tumor growth curves were plotted following the estimation of the tumor mass with the help of histograms [16].

### Luciferase reporter assay

Bioinformatics analysis was performed through LncBASE online database. Afterwards, LINC01410 fragments comprising miR-4319-binding sites (mutant and wild type) were ligated to the pmirGLO Reporter plasmid and transfected to the T24 and 5637 cells. Luciferase Reporter System (Promega, USA) was used to measure luciferase activities following the manufacturer’s guidelines. Firefly luciferase activity was normalized to Renilla luciferase activity [16].

### RNA immunoprecipitation (RIP) assay

Millipore EZ-Magna RIP kit (Millipore, Billerica, MA, USA) was used to conduct RIP-RT-qPCR assay. T24 and 5637 cells lysate was prepared with RIP buffer after incubation with anti-Ago2 and anti-IgG. Following immunoprecipitation, purified RNA was subjected to reverse-transcription which was confirmed by RT-qPCR analysis [15].

### RNA pull-down assay

For RNA pull-down assay, LINC01410 were biotin labeled and subjected to incubation with T24 and 5637 cell lysate for 24 h. After incubation, cellular lysate was further incubated with RNA-bound beads and purified mRNAs were analyzed through RT‐qPCR [15].

### Data analysis

Data from the experiments were subjected to statistical analysis using GraphPad Prism (GraphPad, USA) and SPSS 20.0 (IBM Corp. USA). Differences within the groups and multiple groups were analyzed through Tukey's post-hoc test, ANOVA, and Student’s T-test. Correlations were analyzed through Pearson's correlation analysis. Whereas, Kaplan–Meier analysis was used for survival curves.

## Results

### LINC01410 is over-expressed in BC cells and tissues

**Fig. 1:**
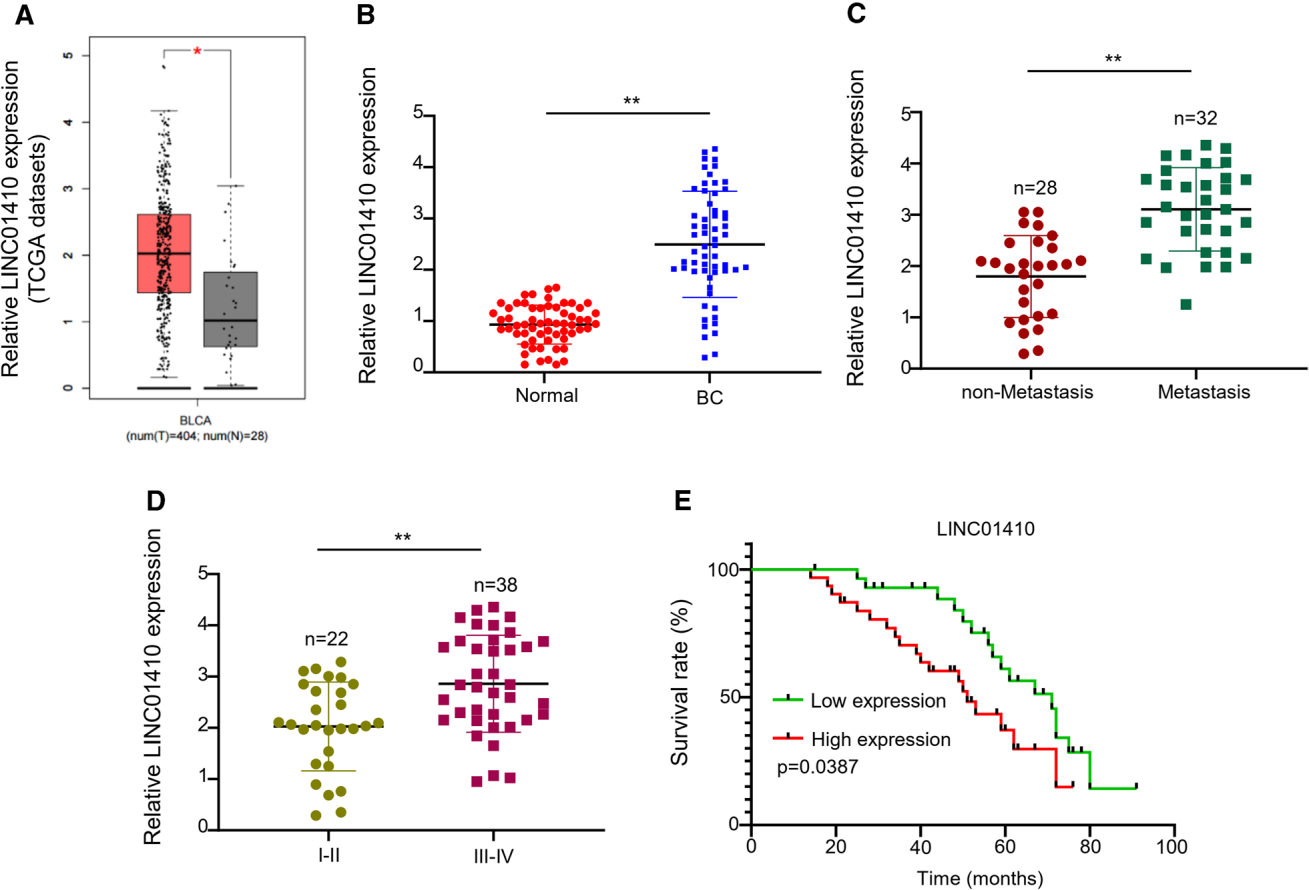
LINC01410 is over-expressed in BC cells and tissues. **A** Analysis of TCGA database showing LINC01410 in BC. **B** qRT-PCR indicating LINC01410 expression levels in BC cells. **C** qRT-PCR indicating LINC01410 expression levels in non-metastasis and metastasis samples. **D** qRT-PCR indicating LINC01410 expression levels at different stages of BC. **E** KM-plotter analysis showing the correlation between LINC01410 and patient’s survival. **P < 0.01

Results of TCGA database indicated a high expression of LINC01410 in bladder cancer (BC) development. Data demonstrated a significant increase of LINC01410 in BC tissues (n = 404) as compared to control (n = 28, Fig. 1A, P < 0.0001) samples. Moreover, results from qRT-PCR displayed remarkedly increased levels of LINC01410 in BC tissues as compared to normal ones (P < 0.001) of BC patients (Fig. 1B). Furthermore, qRT-PCR analysis showed expression levels of LINC01410 in non-metastasis samples (n = 28) and metastasis samples (n = 32) exhibited a significantly higher expression of LINC01410 in the tissues with metastasis of bladder cancer (P < 0.001, Fig. 1C). When compared with expression at different stages of BC, LINC01410 levels were significantly high in BC cells (P < 0.001, Fig. 1D) at stage III-IV (n = 38) as compared with stage I-II (n = 22). The survival curve of BC patients plotted using KM-plotter suggested poor prognosis in patients with high LINC01410 expression (Fig. 1E), showing the crucial role of LINC01410 in BC development.

### Base-tapping LINC01410 inhibited the proliferation, migration, invasion and EMT of bladder cancer cells

**Fig. 2:**
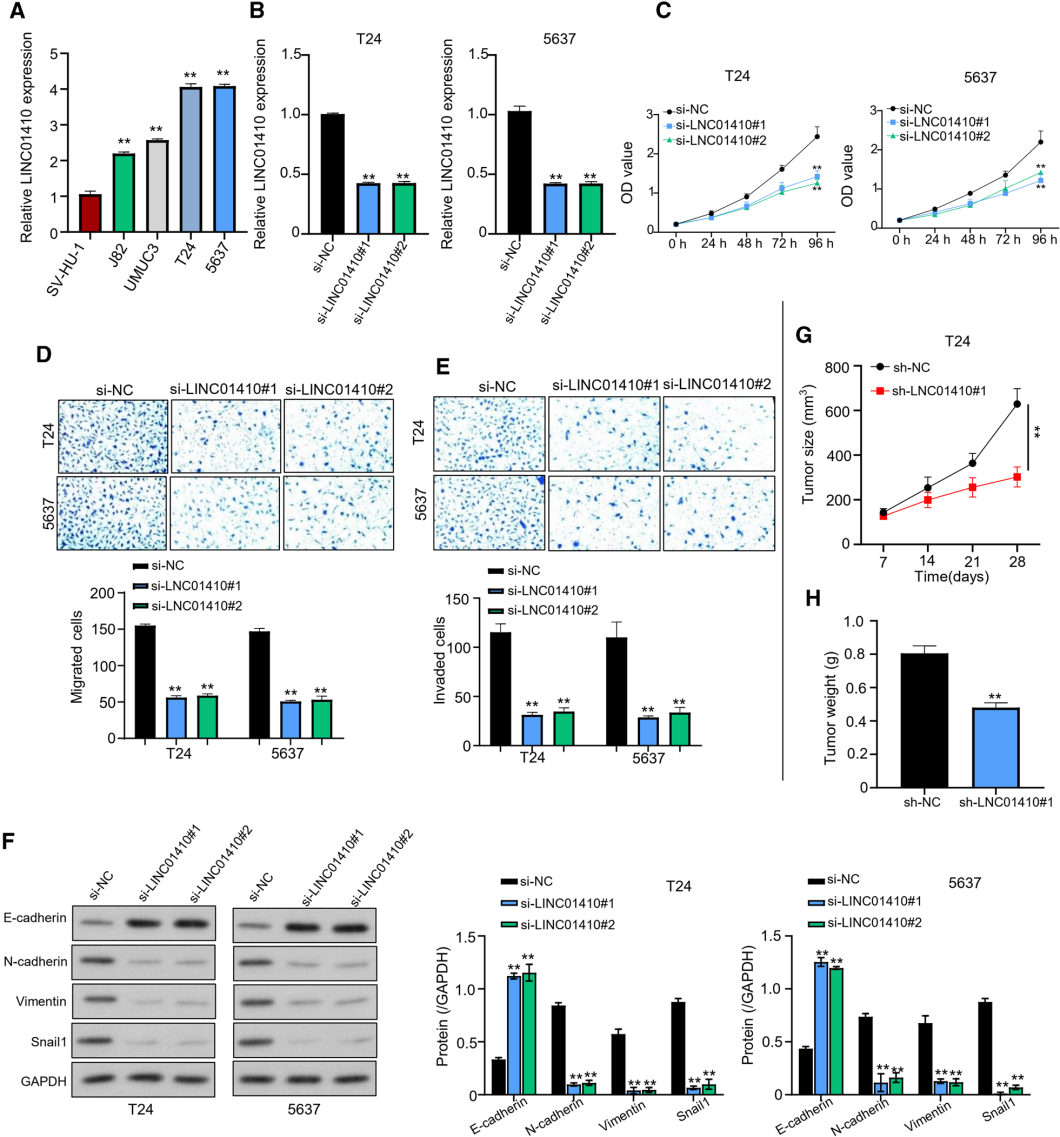
Base-tapping LINC01410 inhibited the proliferation, migration, invasion and EMT of bladder cancer cells. **A** qRT-PCR showing LINC01410 expression levels in BC cell lines includingT24, J82, UMUC3, and 5637, and normal cell line (SV-HU-1). **B** LINC01410 expression levels detected by qRT-PCR when si-RNA transfected in T24 and 5637 in cells. **C** CCK-8 assay for the detection of light absorption values of T24 and 5637 cells. **D** Transwell assay detecting the migration ability of T24 and 5637 cells. **E** Invasion ability of T24 and 5637 cells inoculated with si-NC, si- LINC01410 #1, #2. **F** Western Blot results to detect protein levels of Snail1, E-cadherin, N-cadherin, and Vimentin in T24 and 5637 cells. **G** Subcutaneous tumor xenograft assay in nude mice to detect tumor growth. **H** Subcutaneous tumor xenograft assay in nude mice to detect tumor mass. **P < 0.01

Expression levels of LINC01410 on BC cell lines includingT24, J82, UMUC3, and 5637, and normal cell line (SV-HU-1) were detected by qRT-PCR. Notably, LINC01410 expression levels were significantly upregulated (P < 0.01) in both T24 and 5637 BC cell lines as compared to other BC cell lines and normal SV-HU-1 cell line (Fig. 2A). The two BC cell lines with the highest LINC01410 expression (T24 and 5637) were selected for knockdown (Si-LINC01410 #1, Si-LINC01410 #2) and the knockdown efficiency was detected by qRT-PCR. When compared with loss and gain of function, the knockdown efficiency of si-RNA transfected by T24 and 5637 BC cell two cells was significantly higher (P < 0.01) than that of Si-NC, demonstrating the role of elevated LINC01410 levels in BC development (Fig. 2B). Results of CCK-8 assay highlighted a significant (P < 0.01) reduction in light absorption values at 450 nm by LINC01410, suggesting that LINC01410 knockdown suppressed proliferation (Fig. 2C). Correlation between LINC01410 and metastasis, and cell invasion was evaluated through transwell assay. Data suggested that LINC01410 knockout markedly reduced invasion and migration ability of T24 and 5637 cells (P < 0.01) as compared with the control group, showing that LINC01410 is an oncogenic lncRNA in BC (Fig. 2D, E). Western blot analysis detected protein levels in T24 and 5637 cell lines consisting of si-NC (Negative Control), and si-LINC01410 #1, #2 (experimental groups). The results showed that silencing of LINC01410 considerably reduced expression levels of Snail1, Vimentin, and N‐cadherin which are considered as epithelial and mesenchymal markers. Likewise, E-cadherin expression levels impaired by LINC01410 were significantly (P < 0.01) restored by silencing RNAs; si-LINC01410 #1 and si-LINC01410 #2 (Fig. 2F). Subcutaneous inoculation of T24 cells in control and experimental groups resulted in palpable tumor formation in nude mice after 28 days of implantation. Data from tumor growth curves and histograms demonstrated significantly reduced tumor size and mass in the mice inoculated with LINC01410 knockouts in comparison to control groups with statistical significance of P < 0.05 (Fig. 2G, H, Additional file 1: Figure S1A, B).

### LINC01410 targets miR-4319

**Fig. 3:**
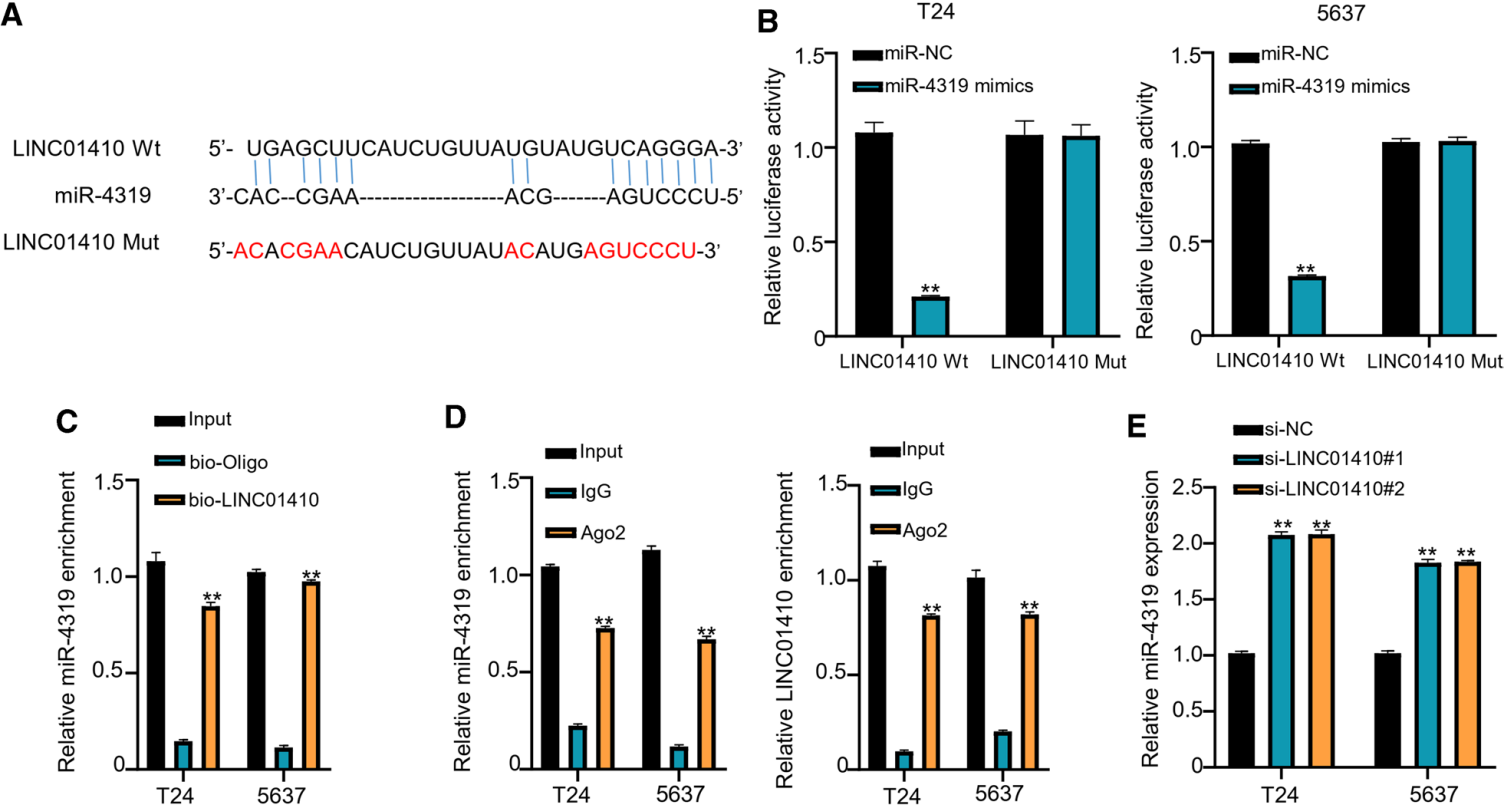
LINC01410 targets miR-4319. **A** Analysis of LncBASE to detect binding sites of miR-4319 in LINC01410. **B** luciferase reporter assay to analyze the activity of LINC01410-WT or MUT reporter in T24 and 5637 cells. **C** RNA pull-down experiment using biotin-labeled LINC01410 probe to show the direct attachment of LINC01410 with miR-4319 in T24 and 5637 cells. **D** RIP-qRT-PCR analysis indicating enrichment of LINC01410 and miR-4319 by Ago2. **E** qRT-PCR results to detect miR-4319 expression levels after LINC01410 knockdown. **P < 0.01

Potential binding elements of miR-4319 in LINC01410 were identified through LncBASE online database (Fig. 3A). Following this, the transfection efficiency of miR-4319 mimics was confirmed through luciferase reporter gene assay. Data documented that miR-4319 overexpression could inhibit the luciferase activity of wild-type LINC01410-WT reporter and vice versa in both T24 and 5637 cell lines, and the inhibition effect disappeared after mutation of the predicted binding site of miR-4319 (Fig. 3B). RNA pull-down experiment using biotin-labeled LINC01410 probe confirmed that LINC01410 directly acted with miR-4319 in T24 and 5637 cells and pulled down more miR-4319 (P < 0.01) as compared to oligo probe (Fig. 3C). Furthermore, results from RIP experiment revealed that LINC01410 and miR-4319 were precipitated in the Ago2 group instead of in the IgG group (P < 0.01, Fig. 3D). The results suggested that knocking down LINC01410 in T24 and 5637 cells significantly upregulated the expression of miR-4319 (P < 0.01, Fig. 3E).

### Overexpression of miR-4319 inhibited the cell proliferation, migration, invasion and EMT of bladder cancer cells

**Fig. 4:**
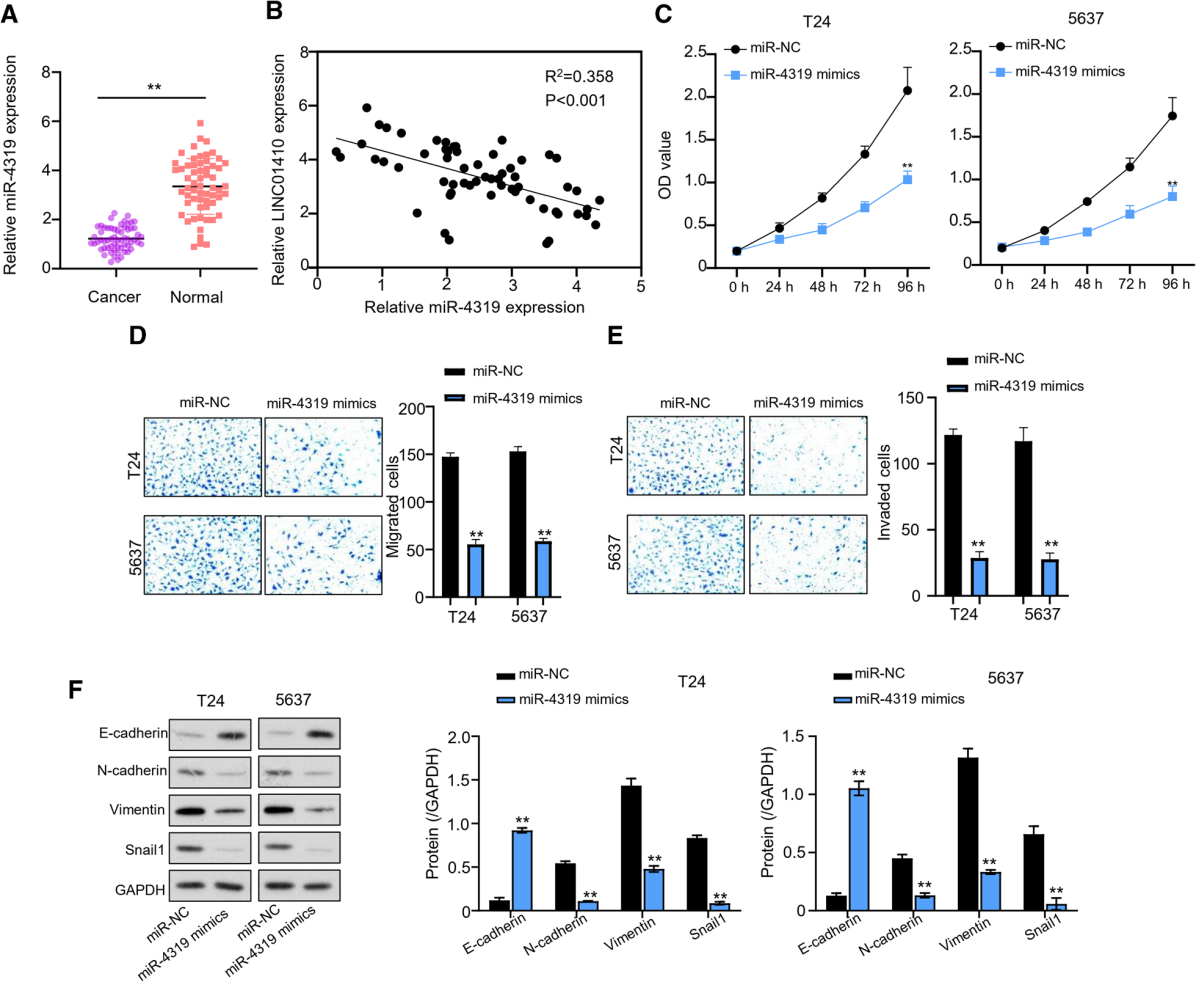
Overexpression of miR-4319 inhibited the cell proliferation, migration, invasion and EMT of bladder cancer cells. **A** QRT-PCR to detect expression levels of miR-4319 in 60 pairs of BC paracancerous tissue and normal tissues, **P < 0.001. **B** Spearman correlation coefficient analysis for the correlation between LINC01410 and miR-4319, **P < 0.001. **C** CCK-8 method performed with miR-4319 to check the light absorption values of T24 and 5637 cells, **P < 0.01. **D** Transwell assay to detect the migratory capacity of T24 and 5637 cells transfected with miR-4319, **P < 0.01. **E** Invasiveness of T24 and 5637 cells transfected with miR-4319, **P < 0.01. **F** Western blot analysis of T24 and 5637 cells transfected with miR-4319, **P < 0.01

qRT-PCR was performed to further explore the effects of miR-4319 expression levels in 60 pairs of paracancerous and normal BC tissues. Data revealed that expression levels of miR-4319 in BC were significantly reduced with a statistical significance of P < 0.001 (Fig. 4A). Moreover, when analyzed through Spearman correlation coefficient, LINC01410 and miR-4319 expression showed a significant negative correlation, and the difference was statistically significant (P < 0.001). Figure 4B illustrates the decreasing trend in miR-4319 expression with a relative increase in LINC01410 expression levels. Next, the light absorption values of BC cell lines groups T24 and 5637 were measured at 0 h, 24 h, 48 h, 72 h, and 96 h of incubation using CCK-8 method. Results from the experiment suggested that transfection with miR-4319 mimics significantly (P < 0.01) reduced the light absorption values in both cell lines as compared to that with miR-NC (Fig. 4C). Subsequently, results from transwell assay documented that transfection with miR-4319 significantly (P < 0.01) reduced the migration ability of T24 and 5637 cells (Fig. 4D). Similarly, the invasion ability of T24 and 5637 cells in different groups (miR-NC and miR-4319) was detected by transwell bioassay performed with matrix glue. The results demonstrated that the invasion ability in miR-4319-transfected T24 and 5637 cells (Fig. 4E) was substantially reduced (P < 0.01). Next, western blot experiments elucidated the effects of miR-4319 transfection on protein levels of Snail1, E-cadherin, N-cadherin, and Vimentin in T24 and 5637 cells of different groups (miR-NC and miR-4319). Data showed that transfection with miR-4319 reduced the Snail1, N-cadherin, and Vimentin levels, while E-cadherin levels were increased (Fig. 4F).

### MiR-4319 targeting Snail1

**Fig. 5:**
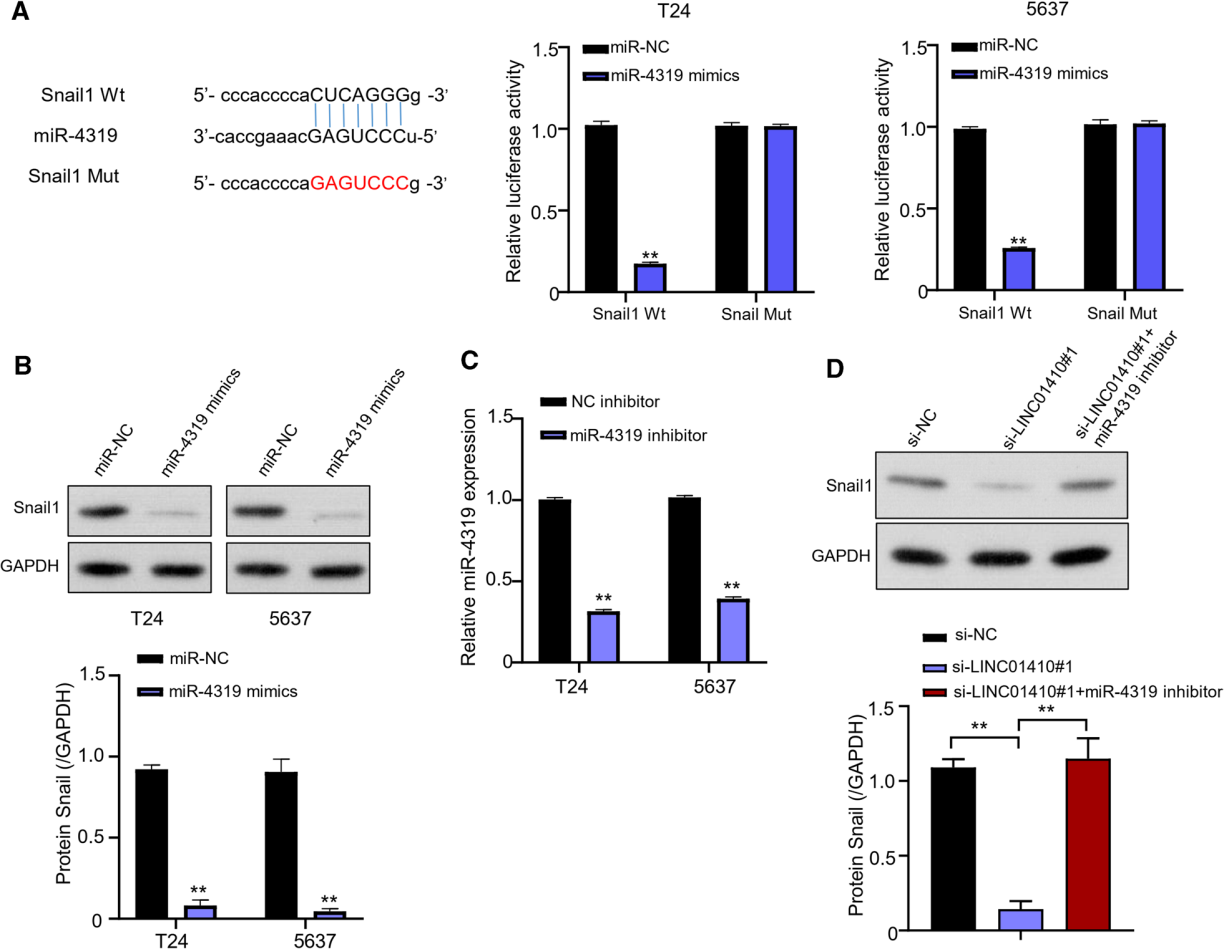
MiR-4319 targeting Snail1. **A** Analysis of StarBASE to detect binding sites of miR-4319 in 3 'non-coding region of Snail1. **B** western blot analysis to detect the expression levels of Snail1 protein in T24 and 5637 cells transfected with overexpressed miR-4319. **C** expression levels of miR-4319 in T24 and 5637 cells transfected with miR-4319 inhibitor, **P < 0.01. **D** western blot analysis to detect protein expression levels of Snail1 in protein in T24 and 5637 cells transfected with si-NC (negative control), si- LINC01410 #1, Si-LINC01410 #1 + miR-4319 inhibitor

Starbase software predicted that 3'-UTR site of Snail1 could be the possible target of miR-4319 and the results were verified through luciferase reporter gene experiments. Results from luciferase reporter assay reinforced that T24 and 5637 cells transfected with miR-4319 inhibited luciferase activity in the cells. Whereas, inhibition effect completely disappeared after mutation of the predicted Snail1 3'-UTR binding site (Fig. 5A). In addition, results from western blot analysis determined that overexpression of miR-4319 significantly (P < 0.01) downregulated the expression of Snail1 protein (Fig. 5B). Similarly, qRT-PCR elucidated that transfection with miR-4319 inhibitors successfully inhibited the miR-4319 expression in both, T24 and 5637 cells (P < 0.01, Fig. 5C). Also, the western blot analysis was used to determine protein expression level of Snail1 in T24 and 5637 cells of various groups including si-NC, si-LINC01410 #1, si-LINC01410 #1 + miR-4319 inhibitor. It showed that LINC01410 decreased the protein expression level of Snail1. Whereas, significant increase (P < 0.01) in Snail1 expression levels were detected with co-transfection of si-LINC01410 #1 + miR-4319 inhibitor (Fig. 5D).

### LINC01410 regulates the progression of bladder cancer cells through miR-4319/Snail1 axis

**Fig. 6:**
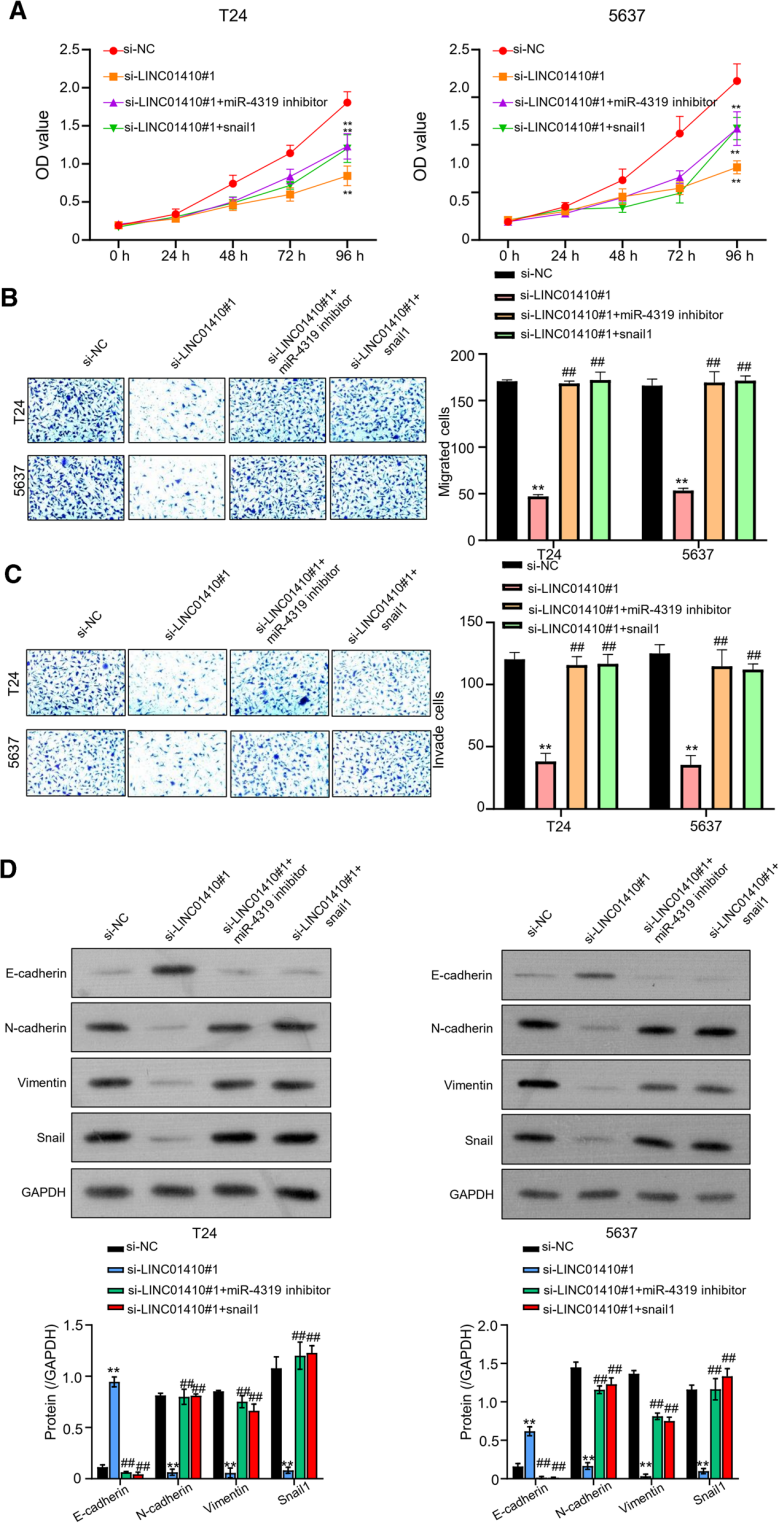
LINC01410 regulates the progression of bladder cancer cells through miR-4319/Snail1 axis. **A** Results from CCK-8 bioassay to investigate the light absorption values of T24 and 5637 cells inoculated with si-NC, si- LINC01410 #1, Si-LINC01410 #1 + miR-4319 inhibitor, **P < 0.01. **B** Transwell assay to detect the migration ability of T24 and 5637 cells co-transfected with miR-4319 inhibitor and Snail1, **P < 0.01. **C** detection of invasion ability of T24 and 5637 cells co-transfected with miR-4319 inhibitor and Snail1, **P < 0.01. **D** western blot analysis to determine the levels of different proteins in T24 and 5637 cells co-transfected with miR-4319 inhibitor and Snail1, **P < 0.01

The role of LINC01410 in bladder cancer cells progression through miR-4319/Snail1 axis was explored by CCK-8 assay. The results showed that transfection of T24 and 5637 cells with si-LINC01410 alone reduced the light absorption values significantly. Nonetheless, when T24 and 5637 cells were co-transfected with miR-4319 inhibitor or Snail1, the light absorption value of cells at 450 nm was (P < 0.01) partially increased (Fig. 6A).

Following the results of CCK-8 assay, transwell bioassay (without matrix glue) was conducted to detect the migratory ability of T24 and 5637 cells in different test and control groups including si-NC (negative control), si-LINC01410 #1, si-LINC01410 #1 + miR-4319 inhibitor, and si-LINC01410 #1 + Snail1. Results showed a clear reduction in migration ability of T24 and 5637 cells transfected with si-LINC01410. Similarly, a significant vice versa trend was observed in both cells when co-transfected with si-LINC01410 #1 + miR-4319 inhibitor, and si-LINC01410 #1 + Snail1 (Fig. 6B). Next, the invasion ability of T24 and 5637 cells in all groups was evaluated through transwell assay. A similar trend was observed in the invasion ability of T24 and 5637 cells in different groups when transwell assay was performed with matrix glue. Results demonstrated that LINC01410 knockout significantly reduced the invasion ability of T24 and 5637 cells. Whereas, co-transfection with miR-4319 inhibitor Snail1 partially increased cell invasion ability of T24 and 5637 cells (P < 0.01, Fig. 6C). Finally, western blot analysis showed that Snail1, N-cadherin, and Vimentin levels were reduced, while E- cadherin levels were increased. However, when T24 and 5637 cells were co-transfected with miR-4319 inhibitor and Snail1, the levels of N-cadherin and Vimentin protein were partially increased, while the levels of E-cadherin protein were partially decreased (P < 0.01, Fig. 6D).

## Discussion

LncRNAs elevated expression in tumor development and the linkage between lncRNAs and cancer progression has become the center of attention in BC tumorigenic mechanisms [25]. Known for their upregulation in protein expression pathways leading to functional disorder in humans, lncRNAs are also targeted as potential therapeutic agents [26]. Several research studies have highlighted the significant expression levels of lncRNA in gastric cancer, cervical cancer, and rectal cancer. However, its role in BC development has not been evaluated before. For instance, lncRNA SOX21-AS1 has been evaluated for its role in the development of lung adenocarcinoma [27]. lncRNA PVT1 has been demonstrated for upregulating SOX2 levels (sex-determining region Y box) and critically propagating breast cancer [28]. Likewise, LINC01410 has been elucidated in gastric cancer metastasis and development by miR-532 [25]. Similarly, it was demonstrated that lncRNA-XIST could promote BC metastasis by sponging miR-124 [29]. The key observations of the current study highlight the LINC01410 expression levels in BC tissues and cell lines. F. First of all, data obtained from TCGA database showed upregulated levels of LINC01410 in BC. Results from CCK-8 assays demonstrated that LINC01410 knockdown considerably repressed BC cell growth *in-vitro*. Moreover, Transwell assay demonstrated the markedly repressed invasion of BC cells. Kaplan Meyer analysis and association analysis have highlighted the *in-vivo* potential of LINC01410 as an oncogene which was further validated through a xenograft nude mouse experiment.

It was reported that the miR-4319, as a cancer related miRNA, could induce inhibition of EMT and prevente cancer stemness of hepatocellular carcinoma (HCC) through targeting FOXQ1 [22]. Similarly, miR-4319 has been shown to impair the malignancy in breast cancer by self-regulation and tumorigenesis of stem cells [20]. Recently more studies indicated the potential role of miR-4319 in inhibiting thyroid cancer by controlling FUS‐stabilized SMURF1 [21]. Nevertheless, the role of miR-4319 in BC has not been shown before. In this study, LINC01410 directly targeted the miR-4319, suggesting LINC01410 as a potential prognostic biomarker for BC. To further verify the role of miR-4319 in BC, in vitro functional experiments were established. Analysis of miR-4319 in BC tissues elucidated low levels. Whereas, inhibitory effects of miR-4319 mimics on BC cell growth and invasiveness were maintained by LINC01410 upregulation, and the tumor suppressor effect of miR-4319 was consistent with that of other studies [22, 30, 31].

EMT is orchestrated by a set of EMT-activating transcriptional factors (EMT-TFs), whose core set includes Snail1 (Snail), Snail2 (Slug), Twist1, Zeb1, and Zeb2 [32]. Since Snail1 was characterized in 2000 as a transcriptional repressor and an inducer of EMT and invasion in tumor cells, it has been the subject of study for many cancer biologists. For instance, Snail1-dependent p53 repression regulates the expansion and activity of tumour-initiating cells in breast cancer [33], CDK4/6-dependent activation of DUB3 regulates cancer metastasis through Snail1 [27]. In our study, mechanistic insights into miR-4319 function predicted Snail1 as the direct target of miR-4319, suggesting that Snail1 was a possible promoter of the invasiveness and angiogenesis of BC cells as previous research found [34–36]. As per our best knowledge, the present study is the preliminary report on LINC01410 as the promoter of migration, invasion, and EMT of bladder cancer cells through up-regulating Snail1 protein by sponging miR-4319. Lastly, suppression of miR-4319 markedly abolished the repressive effects of knocking down of LINC01410 on proliferation and invasion of BC cells, confirming that it was a tumor suppressor in BC. Nonetheless, downstream signaling of LINC01410/miR-4319 axis still needs to be explored and validated.

## Conclusions

In conclusion, the study showed that LINC01410 is highly expressed in bladder cancer and is closely related to the metastasis of BC. Mechanistically, LINC01410 promoted the cell proliferation, migration, invasion, and EMT transformation of BC cells through the up-regulation of Snail1 protein by sponging miR-4319.

## Supplementary Information

**Additional file 1: Figure S1.** Base-tapping LINC01410 inhibited tumor growth of 5637 in vivo. (A) subcutaneous tumor xenograft assay in nude mice to detect tumor growth. (B) subcutaneous tumor xenograft assay in nude mice to detect tumor mass. **P < 0.01.

## Data Availability

All supporting data of this work, which are not available in public because of the ethical restrictions are available from the corresponding author upon request.
