# Supplementary figures and images for: LINC01410 leads the migration, invasion and EMT of bladder cancer cells by modulating miR-4319 / Snail1
Source: Cancer Cell Int. 2021 Aug 14;21:429. doi: 10.1186/s12935-021-02119-z (PMC8364693; doi:10.1186/s12935-021-02119-z)

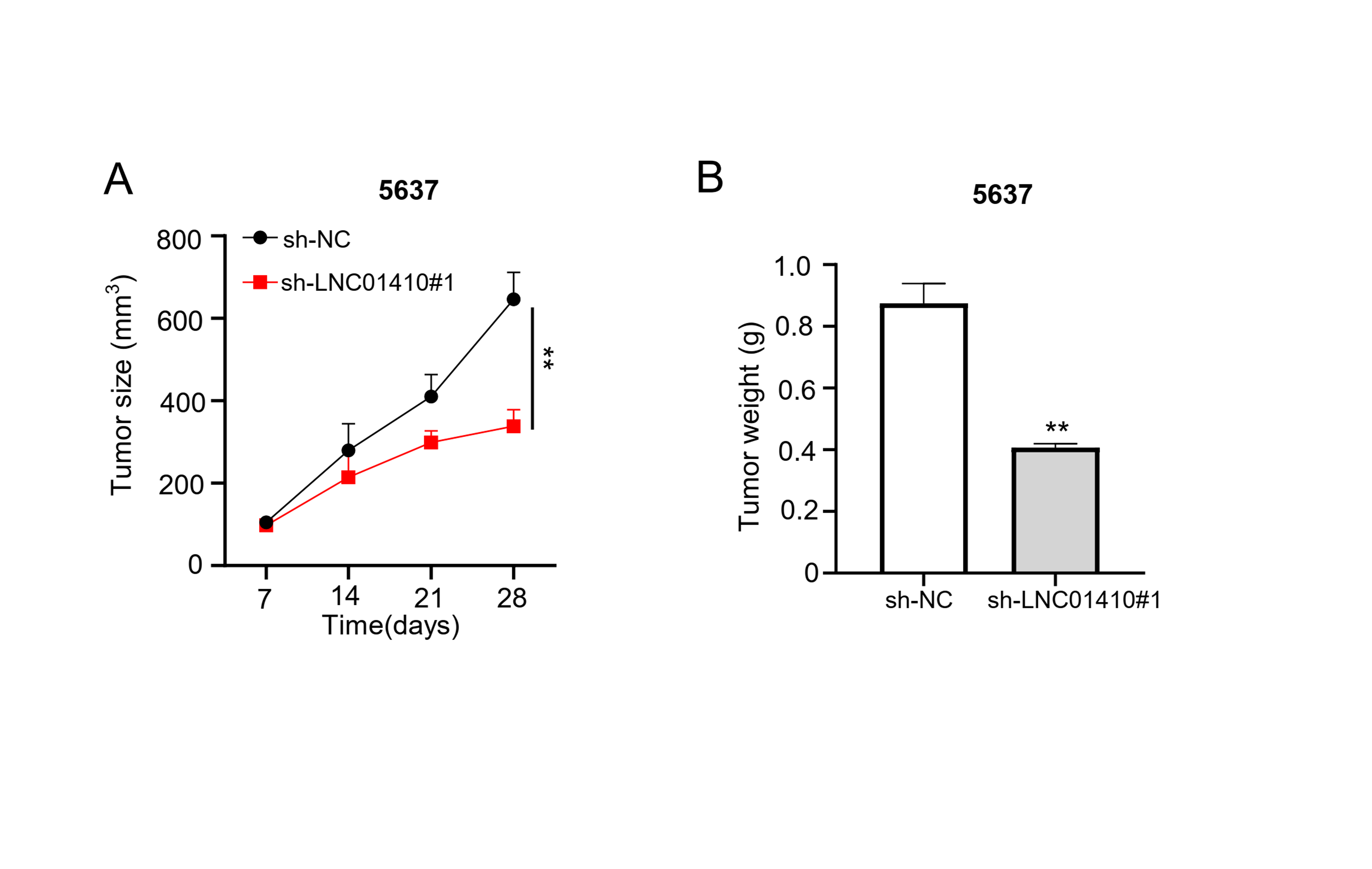

Supplement: Supplementary file 1 — Additional file 1: Figure S1. Base-tapping LINC01410 inhibited tumor growth of 5637 in vivo. (A) subcutaneous tumor xenograft assay in nude mice to detect tumor growth. (B) subcutaneous tumor xenograft assay in nude mice to detect tumor mass. **P < 0.01. [file 12935_2021_2119_MOESM1_ESM.tif]
